# Assessment of the Potential of Wrist-Worn Wearable Sensors for Driver Drowsiness Detection

**DOI:** 10.3390/s20041029

**Published:** 2020-02-14

**Authors:** Thomas Kundinger, Nikoletta Sofra, Andreas Riener

**Affiliations:** 1 AUDI AG, 85045 Ingolstadt, Germany; nikoletta.sofra@audi.de; 2 Faculty of Computer Science, Technische Hochschule Ingolstadt (THI), 85049 Ingolstadt, Germany; andreas.riener@thi.de; 3 Department of Computer Science, Johannes Kepler University (JKU), 4040 Linz, Austria

**Keywords:** drowsiness detection, driver state, simulator study, physiological measures, machine learning, wearable sensors, automated driving

## Abstract

Drowsy driving imposes a high safety risk. Current systems often use driving behavior parameters for driver drowsiness detection. The continuous driving automation reduces the availability of these parameters, therefore reducing the scope of such methods. Especially, techniques that include physiological measurements seem to be a promising alternative. However, in a dynamic environment such as driving, only non- or minimal intrusive methods are accepted, and vibrations from the roadbed could lead to degraded sensor technology. This work contributes to driver drowsiness detection with a machine learning approach applied solely to physiological data collected from a non-intrusive retrofittable system in the form of a wrist-worn wearable sensor. To check accuracy and feasibility, results are compared with reference data from a medical-grade ECG device. A user study with 30 participants in a high-fidelity driving simulator was conducted. Several machine learning algorithms for binary classification were applied in user-dependent and independent tests. Results provide evidence that the non-intrusive setting achieves a similar accuracy as compared to the medical-grade device, and high accuracies (>92%) could be achieved, especially in a user-dependent scenario. The proposed approach offers new possibilities for human–machine interaction in a car and especially for driver state monitoring in the field of automated driving.

## 1. Introduction

In its “2025 Roadmap”, the EuroNCAP (European New Car Assessment Programme) states that driver state monitoring will be part of the safety assessments by 2020 and listed in the category of primary safety [1]. Around 90% of all accidents are based on human errors [2], and a considerable part of them occur due to drowsiness. In the USA, for example, 21% of fatal crashes can be attributed to drowsiness [3]. The risk of an accident is almost four times higher when being in a drowsy physical state [2]. Therefore, reliable driver state monitoring systems are required. By warning an impaired driver already in an early stage, these systems have high potential to increase road safety [1]. Automated driving is expected to have an even higher impact on increasing road safety, as it eliminates potential driver errors. However, the risk factor drowsiness plays and will continue to play a crucial role until the complete rollout of advanced automated driving technologies is achieved. Utilizing the definition of the automation of the driving task provided by the Society of Automotive Engineers (SAE), level 1 (supported driving), 2 (partial automation), and 3 (conditional automation) specifically demand a method to observe the driver’s state reliably [4]. In level 1, the driver is supported by advanced driver assistance systems (ADAS) that take over either longitudinal or lateral vehicle control in certain situations. In level 2, the driver is required to monitor the system continually and to have the capacity to intercede in a sufficient time period. In level 3, the driver is excluded from monitoring the system yet needs to react sufficiently to a take-over request anytime. At these levels, the driver acts as a fallback for the automated system. However, the decreasing active involvement in driving increases the risk of getting drowsy faster.

## 2. Driver Drowsiness Measurement Technologies

For driver drowsiness detection, various systems and measurement technologies have already been developed that can be summarized into subjective, behavioral, vehicle-based, as well as physiological measures [5,6]. Apart from the measurement of drowsiness in an automotive context, its detection also plays a crucial role and finds use in other areas, such as in the field of aviation or marine operations. Therefore, some examples from other areas with similar detection approaches are listed.

Subjective measures are mainly used as a reference metric for obtaining a definition and ground truth for drowsiness [7,8]. These measures include self- and observer ratings. For driver self-ratings, questionnaires such as the Karolinska Sleepiness Scale (KSS) [9], the Epworth Sleepiness Scale (ESS) [10], the Stanford Sleepiness Scale (SSS) [11], or the Visual Analog Scale (VAS) [12] are provided. For observer ratings, experts or trained raters observe the driver either in real-time [13] or by watching videos recorded during an experiment [14]. They evaluate the driver’s current drowsiness state with scales that focus on sleep-induced indicators in the facial region [7,8,14,15]. Since this type of measurement depends either on the driver himself/herself or an external observer, the usage in a real-world driving scenario is not possible.

Behavior-based methods observe the driver’s face with a driver monitoring camera to automatically detect early signs of drowsiness utilizing image processing. This technique has already been investigated for a long time [16,17]. Particularly advantageous is their non-intrusiveness, which makes their usage inside a vehicle very customer friendly. Due to advancements in camera technology in combination with novel approaches in computer vision and image processing, camera-based drowsiness detection has been receiving more and more attention in recent years [18]. These methods evaluate mainly three parameters: eye movements (eye blinking and eye closure activity) via eye-tracking, that was also investigated for usage in maritime operations and aviation [19,20,21], facial expressions (yawning, jaw drop, brow rise, and lip stretch), and head position (head scaling/nodding) [22]. In particular, many studies focused on the use of machine (deep) learning-based approaches [23,24,25,26,27]. Apart from research, numerous commercial products are available that rely on behavioral measures for drowsiness detection. These range from camera-based methods [28,29] to devices worn behind the ear [30] or glasses [31], that also found their way into the aircraft [32]. However, differing blink frequencies and amplitudes from individual to individual can impact the quality of the observing framework [33]. Moreover, insufficient lighting, sunglasses, or partial visibility of the head by, for example, an unusual sitting position can limit the performance of the monitoring system [22,34].

Vehicle-based systems evaluate driving behavior. The most commonly used measures are Steering Wheel Movement (SWM) and Standard Deviation of Lane Position (SDLP) [22]. In the former, unnatural steering behavior induced by drowsiness is detected by using a steering angle sensor. In the latter, the position of the vehicle in the lane is monitored, and the deviation from the centerline of the lane calculated [35,36,37,38,39,40]. These measures are non-intrusive and show high potential in detecting drowsiness. This is also reflected in the fact that well-known car manufacturers have been making use of this technology for several years in their series production cars [41,42,43]. However, their reliability is influenced by driving expertise, geometric, and environmental conditions as well as intoxication [5]. During automated driving, these parameters will be more difficult to evaluate since the automated system controls the vehicle.

The most commonly investigated physiological-based methods for driver drowsiness detection [22] utilize information based on brain activity (electroencephalography (EEG)) [44,45,46], cardiac activity (electrocardiography (ECG)) [47,48,49], ocular activity (electrooculography (EOG)) [50,51,52], and muscle tone (electromyography (EMG)) [53,54]. Physiological measures are reliable, accurate, and show high potential in differing wakefulness and sleep during driving [5,55]. They change in the very early stages of drowsiness compared to behavioral or vehicle-based ones that have the ability to warn the driver in time [5]. However, a major disadvantage is their intrusiveness, e.g., by attaching adhesive electrodes on the subject’s chest or scalp. Therefore, new and less intrusive monitoring strategies for recording this kind of signals are required. Different approaches have been proposed to record them in a less or even non-intrusive way inside a vehicle for driver drowsiness detection. For example, experiments were conducted to measure heart rate or ECG for Heart Rate Variability (HRV) analysis via integrated sensors on the steering wheel [56,57,58]. However, their usage is limited in the context of automated driving since one or even both hands have to touch the steering wheel for a longer time. In the work of Solaz et al., the driver’s breathing rate was captured from real-time image recognition. Results show that the kind of clothes influences and reduces system performance [59]. As part of a research project funded by the European Union, bio-sensors were built into car seat fabrics and seatbelts to measure heart rate and respiration [60]. For the approaches mentioned, it is necessary to incorporate sensors in the vehicle additionally. Moreover, the upgrade of existing vehicles would be associated with considerable effort. Apart from research, also on the market, systems for drowsiness detection with physiological signals are available. These devices measure heart rate and Electrodermal Activity (EDA) with a bracelet [61], only EDA with a double ring [62] or Galvanic Skin Response (GSR) with wristband and ring [63]. Lately, devices from the consumer electronics market are being integrated into the vehicle, which could be a solution to the problems mentioned. In this market, especially the trend of fitness tracking and health monitoring in everyday life with wrist-worn smart wearable devices (e.g., smartwatch, fitness tracker) prevails. Due to their cheapness and the fact that they can be worn comfortably like a watch, their usage is expanding continuously, also demonstrating enhancing customer satisfaction and acceptance. The market for wearable gadgets is consistently growing (two-fold growth from 2015 to 2017). In a very recent report, it was stated that the worldwide smartwatch shipments increased by 56% within 2018 compared to the time period in the previous year [64]. Moreover, it was shown that physiological data from wearables exhibit very high correlations with more advanced measurement devices [65]. Encouraged by the progress in the development of smart wearable devices during the last years, in this work, their suitability in an automotive environment and in particular, in the field of driver drowsiness detection will be investigated. Apart from their use in daily health and fitness monitoring for which these devices were mainly designed, in this way, their scope of use could be further enhanced. This kind of driver-vehicle interaction offers the option of being easily integrated into a vehicle, and existing vehicles can also quickly be upgraded with this technology without having to install additional sensors. Their usage inside a vehicle would enable long-term recording of physiological signals in a non-intrusive and for the driver familiar way.

The rest of the paper is organized as follows: In Section 3, previous work in driver drowsiness detection using wrist-worn wearable devices are discussed, the research gap is highlighted, the contribution, as well as research questions, are presented. Section 4 describes the methodology underlying the work, including simulator study, driver state analysis, feature analysis, data set preparation, and the classification of driver drowsiness. In the results section (Section 5), the findings from feature selection and classification are presented and discussed. This is followed by the conclusion (Section 6), which summarizes the work and core findings.

## 3. Previous Work in Driver Drowsiness Detection Using Wrist-Worn Wearable Devices

Lee et al. utilized the built-in motion sensors of a smartwatch for driver drowsiness detection by evaluating the driving behavior [66]. Twenty subjects participated in a simulator study. Time, spectral, and frequency domain features were extracted and mapped to the subject’s drowsiness self-ratings. A support vector machine (SVM) classifier reached an accuracy of 98.15%. A similar approach was followed in the work of Lee et al. where accelerometer and gyroscope data in combination with a SVM classifier was used, resulting in an accuracy of 98.8% [67]. Leng et al. developed a wristband with a photoplethysmogram (PPG) and galvanic skin response (GSR). From data of 20 subjects, five features were extracted, including HRV and respiratory rate, and labeled with self-ratings [68]. A SVM classifier resulted in an accuracy of 98.30%. In the work of Choi et al., a wrist-worn wearable device with sensors for PPG for HRV analysis, GSR, temperature, acceleration, and gyroscope was developed. Twenty-eight people participated in their simulator study. Labels were gathered by analyzing signs of sleepiness in their facial expressions. With a SVM classifier, an accuracy of 98.43% of accuracy was reached [69]. Lee et al. conducted a simulator study with six participants and combined data from a PPG sensor of a Polar (Polar Electro Oy, Kempele, Finland) smartwatch with ECG data measured with a breast belt. Labels were assigned by evaluating videos of the driver’s face and driving behavior. Their classification in the form of recurrence plots resulted in an accuracy of 70% [47]. The heart rate measurement of a smartwatch was fused with PERCLOS (percentage of eye closure) in the work of Li et al. [70]. A study in a simulated environment with 10 participants was conducted. An accuracy of 83% was obtained with a SVM classifier.

By utilizing data from smart wearables devices for driver drowsiness detection, promising results were achieved in previous works. However, some devices were specially developed for these studies and are not readily available on the consumer electronics market [68,69]. When available on the market, they were combined with another or more intrusive measurement [47,70]. Focusing on future automated driving, sensors like accelerometer or gyroscope will be more difficult or in a different way to use since movement patterns from the steering cannot be continuously evaluated for driver state detection [66,67]. In view of this, the aim of this work was to investigate and assess the potential and feasibility of using physiological data (HRV) from a wrist-worn wearable device, readily available on the consumer electronics market, as single data source in combination with a common machine learning model for driver drowsiness detection. In further steps and based on the results, the knowledge gained and information provided can then also be applied to develop multimodal systems with a sensor fusion approach and to merge the data of the wrist-worn wearable device with other non-intrusive in-vehicle sensors, e.g., a driver monitoring camera. Within this work, the following research questions (RQ) are addressed:

- RQ1: Is it possible to reliably detect driver drowsiness by using physiological data (HRV) from a wrist-worn wearable device as single data source in combination with a machine learning classifier?
- RQ2: Considering the in-vehicle setting, how do the results of the consumer device differ from a more intrusive medical-grade device?
- RQ3: How do the results differ in the case of user-dependent vs. user-independent tests?

## 4. Methodology

**Figure 1:**
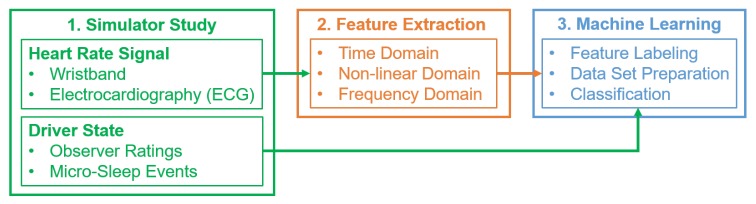
Overview of the methodology for the proposed drowsiness detection system. ECG: Electrocardiography.

To examine the feasibility of our proposed approach for detecting driver drowsiness, the methodology, as presented in Figure 1, was applied. The three main stages, including simulator study, feature extraction, and machine learning, will be described in more detail in the following sections.

### 4.1. Simulator Study

To record measurements of heart rate data and driver state, a user study in a simulator was conducted, which will be explained in the following sections. A more detailed description of the study can be found in the work of Kundinger et al. [71]. The following summary focuses on contextual information and mainly on those parts of the user study that are relevant to this work.

#### 4.1.1. Simulator

**Figure 2:**
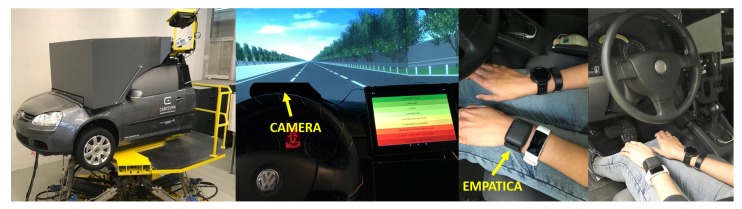
(**Left**) High-fidelity driving simulator (Hexapod system) as used in the study; (**Center**) Study setup with camera mounted on top of cockpit; (**Right**) Wearable devices on wrist of participant.

The experiment was carried out in a high-fidelity driving simulator at Technische Hochschule Ingolstadt (THI, Ingolstadt, Germany). The course chosen was a monotonous track (closed loop with a length of around 13 km) in the form of a three-lane highway with little traffic (see Figure 2). The average temperature in the test vehicle was $25{\;}^{\circ }$C. No radio or music was played during the drive.

#### 4.1.2. Participants

Thirty subjects participated in the study, students, or recruited via an advertisement in a local newspaper. Concerning a report from the Sleep Health Foundation that contains recommendations about sleep requirements over the life span, subjects were divided into two age groups [72]. Fifteen younger (6 female; 9 male; mean (M) = 22.87 years; standard deviation (SD) = 1.81 years) subjects were chosen in the age range 20–25 and 15 older subjects (8 female; 7 male; M = 67.60 years; SD = 1.88 years) in the range 65–70. For their participation, each subject received €35. The participation requirements were a valid driving license, self-reported good health, no sleep disturbances, and no restrictions on driving ability. They were not allowed to consume caffeinated drinks within five hours before the study.

#### 4.1.3. Study Procedure

In the beginning, the subjects got instructions and filled out a questionnaire. The first part contained some basic demographic questions and queried details about sleeping behavior and health. Secondly, subjects were asked to answer the questions of the ESS to measure their daytime sleepiness [10]. This was followed by a 10-min manual test drive to become familiar with the simulator. Then, the 45-min automated ride began. The speed limit for the automated drive was set to 110 km/h. The simulation car traveled with autonomous cruise control (ACC). It automatically accelerated and decelerated, depending on the traffic. A total distance of 73.7 km was covered during the partially automated drive. To reproduce SAE level-2 driving [4], drivers were asked to monitor the driving condition continuously and to be prepared to assume control in the event of a take-over request. In the context of this study, take-overs were not necessary as they would probably have interfered with the development of drowsiness. The request to observe the driving environment had the objective to maintain the driver’s concentration, inducing a more rapid development of drowsiness. Participants were asked not to use their mobile phone, not to eat or drink and abstain from chewing gum, or to nod off. They were not allowed to carry out any secondary tasks. Moreover, the participants drove manually (in randomized order) under similar conditions. The study was conducted at 9 a.m., 1:30 p.m., and 5:30 p.m. An almost equal number of participants were available at each time (morning: 9, afternoon: 11, evening: 10).

The corresponding results of the questionnaires are published and can be found in the work of Kundinger et al. [71].

#### 4.1.4. Measurement Technique

In terms of the wrist-worn wearable consumer device, heart rate signals were recorded utilizing an Empatica E4 wristband (Empatica Inc., Boston, MA, USA) (further referenced as “wristband”) [73]. The Empatica E4 wristband records inter-beat intervals (IBIs), the time between two successive and individual heartbeats, for calculating the instantaneous heart rate that will be later used in HRV analysis. Another commonly used description of the IBIs is RR intervals, which is the time elapsed between two successive R waves, i.e., two heartbeats, of the QRS complex on the ECG. This IBI sequence is received from the PPG/BVP (blood volume pulse) signal with a sampling frequency of 64 Hz. McCarthy et al. checked the validity of the Empatica E4 wristband against clinical standard gears in recognizing the anomalies in a heartbeat and found a comparable data quality of 85% between these devices [74]. A 3-channel ECG measurement device (further referenced as “ECG”), the Faros from Bittium (Bittium Corporation, Oulu, Finland) [75], served as the medical-grade device and reference for the wristband. The ECG data was recorded with the maximum possible sampling frequency of 1000 Hz. With five adhesive electrodes, it was attached to the subject’s upper body. RR intervals were stored for HRV analysis. By performing HRV analysis on ECG or PPG signals, the activity of the Autonomic Nervous Systems (ANS) can be obtained [76]. This activity alters with stress and drowsiness [77]. The two major components of the ANS are the sympathetic and parasympathetic nervous systems. The decrease of parasympathetic activity and increase of sympathetic activity is associated with the vigilance of a person. The increase in parasympathetic activity and the decrease in sympathetic activity indicates relaxation [77]. The normalized Low-Frequency (LF) band power of HRV follows the dominance of sympathetic activity, whereas High-Frequency (HF) power is associated with parasympathetic activity [78]. Therefore, investigating ANS activity may help to get deeper insights in a driver’s drowsiness [79]. Besides physiological signals, videos of the driver’s face were recorded, which were used to assess the driver’s current drowsiness state. Therefore, a camera was mounted on top of the cockpit and directed towards the driver’s face (see left part of Figure 2).

Further data (not evaluated in this work) were recorded: Physiological data with three additional wearable devices (Polar A370 (Polar Electro Oy, Kempele, Finland), Garmin Vivosmart 3 (Garmin Ltd., Schaffhausen, Switzerland), Garmin Forerunner 235 (Garmin Ltd.)), driving behavior-related parameters and drowsiness self-ratings (see Kundinger et al. [71]).

### 4.2. Driver State Analysis

In order to obtain a reliable and valid ground truth of drowsiness for supervised machine learning, a two-stage process with a combination of video ratings and image processing was applied.

#### 4.2.1. Video Ratings

Observer ratings of the driver’s facial expressions and behaviors were collected offline after the study. The 45-min partially automated ride was split into nine intervals of five min length. From each 5-min interval, one minute was extracted to be rated by the observer. Sandberg et al. found that most indicators of driver sleepiness can be observed for intervals of 60 s or longer to obtain reasonable signs of a driver’s sleepiness state [80]. The order of the extracted segments of one minute length was randomized per participant with the aim of eliminating the time dependency of the single segments. Video segments at the end of a participant’s drive would probably be rated higher compared to ones in the beginning. To increase the reliability of the results, two trained individuals rated all videos separately. Following that, the segments with inconsistent ratings were evaluated and discussed by both raters, and a joint rating was set. The obtained rating of the extracted minute from the respective 5-min interval represents the entire interval with the assumption that the drowsiness state does not change abruptly, but rather more slowly. Weinbeer et al. published the scale that was used for the observer ratings. This scale categorizes drowsiness into six levels ranging from level 1 (not drowsy) to level 6 (extremely drowsy) (see Table 1) [13].

Taking into account the 30 subjects and the 45-min partially automated ride, a total of 270 min, i.e., ratings, would have been available for evaluation. However, for some subjects, problems with the video recording occurred, or the face was only partially visible in the video, for example, due to an unusual seating position. These segments were removed so that in the end, 244 min were evaluated. By both raters, the same decisions were made in 191 of 244 cases, which correspond to a percentage of 78.28%. Inter-rater reliability in the form of Cohen’s Kappa resulted in a value of 0.69, which represents “substantial agreement” following the classification of Landis and Koch [81].

#### 4.2.2. Detection of Micro-Sleep Events through Image Processing

In addition to the observer ratings, the recorded video data was evaluated through image processing. As the raters only assessed one minute in each 5-min interval of the 45-min ride, drowsiness indicators in the remaining four minutes could not be considered for the final rating. Concerning the drowsiness scale, this would have been particularly critical if a micro-sleep event had taken place within the non-rated minutes since levels 4 to 6 include the specific eyelid closure time as a drowsiness indicator. Therefore, all detected events with an eye closure of at least one second or longer were used, as well as the respective eyelid closure duration in order to assign the appropriate level of drowsiness. With this additional step, the observer ratings could be cross-checked and enhanced.

In total, 201 micro-sleep events were detected for 14 out of 30 subjects. All events detected were manually double-checked based on the corresponding frame numbers in the video file. The events were split, as shown in Table 2 to be assigned directly to drowsiness levels 4 to 6 on the scale used. As the scale (see Table 1) does not take into account eyelid closures between 3 and 4 s (level 5: 2–3 s and level 6: 4 s or more), these have been added to level 5 (see Table 2).

After receiving video ratings and micro-sleep events, both measures were combined. For each subject, the ratings for the 5-min intervals with micro-sleep events were adjusted if necessary. A change in the drowsiness level was done for 23 out of the 244 received ratings. The drowsiness level was corrected 20 times upwards and one time downwards. Two new ratings could be gained, giving a total of 246 ratings (see Table 3).

Since the main focus of this work is on the assessment of the potential of wrist-worn wearable sensors for driver drowsiness detection, the simple case of binary classification of drowsiness (“not drowsy” vs. “drowsy”) is considered. In further steps and with the knowledge acquired in this work, the classification of sleepiness can also be extended to a multi-class problem. Therefore, the six drowsiness levels of the considered scale were divided into two groups. The “non-drowsy” class contains levels 1 to 3 (not drowsy, slightly drowsy, moderately drowsy) whereas the “drowsy” class covers levels 4 to 6 (drowsy, very drowsy, extremely drowsy). Table 4 shows the distribution of the number of grouped ratings on the two classes before and after the adjustment with micro-sleep events. A class imbalance is apparent.

**Figure 3:**
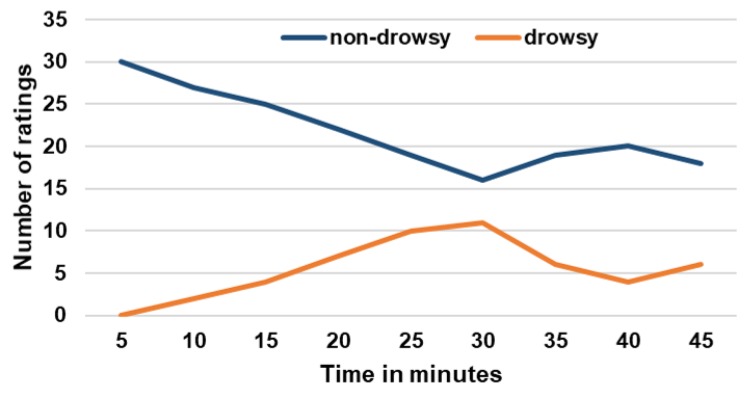
Localization in time of number of ratings for both the “non-drowsy” and “drowsy” class.

In Figure 3, the localization in time of the number of ratings for both the “non-drowsy” and “drowsy” class after the integration of micro-sleep events is presented. This shows that the number of “drowsy” ratings increased almost linearly across all subjects up to a driving time of 30 min. In contrast, the number of “non-drowsy” ratings decreased in the same time interval. From minutes 30 to 40, it shows exactly the opposite, and a trend similar to the one at the beginning of the drive can be observed in the last five minutes. From this it can be deduced that drowsiness increased on average across all subjects up to minute 30, and decreased from minutes 30 to 40. Towards the end, the level of drowsiness rose again.

### 4.3. Feature Extraction and Data Set Preparation

**Figure 4:**
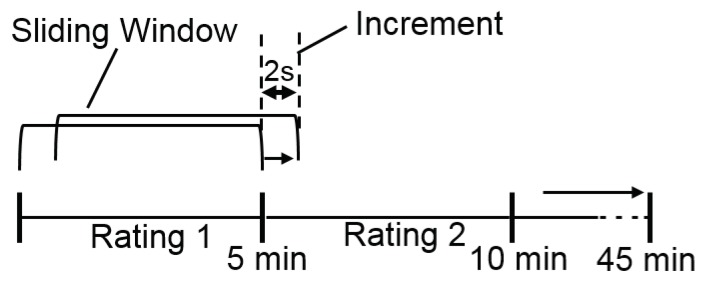
Sliding window approach for feature extraction and labeling exemplary for time intervals 1 and 2.

Feature extraction and data set preparation for machine learning was performed with MATLAB (The MathWorks, Inc., Natick, MA, USA) (version 2018a). The different sampling rates of the two devices were not adjusted because this work aims to compare the usage of data from a consumer and a medical-grade device. Since three channels were recorded with the ECG measuring device, but only one and the same channel was used for all participants for further analysis, the three channels of all subjects were visually inspected in terms of data quality and possible artifacts, e.g., undetected RR peaks in the ECG pattern, with the Kubios HRV analysis software [82]. Finally, the RR peaks of Channel 1 were applied in further analysis in raw format. In terms of Empatica E4 wristband, it should be mentioned that the wristband uses an algorithm during the recording of the data and thus the processing of the PPG/BVP signal that already filters and removes false peaks due to noise (e.g., motion artifacts) [83]. For this reason, the raw data of the wristband was used for the following analyses and is not further filtered or preprocessed. IBIs from wristband and ECG were processed in time, frequency, and non-linear domain for HRV analysis. In several previous works, HRV was applied for driver drowsiness detection [69,76,84]. HRV features were extracted from 5-min windows of the signal with reference to the Task Force of the European Society of Cardiology and the North American Society of Pacing and Electrophysiology that suggests 2–5 min windows for the analysis of frequency-domain features [78]. A sliding window with a 2-s increment was used for generating the feature vectors (see Figure 4). In the frequency domain, Power Spectral Density (PSD) was computed with Lomb–Scargle periodogram, a non-parametric technique that does not require any earlier information of the signal parameters. The advantage of utilizing Lomb–Scargle periodogram over other non-parametric techniques like Welch is that no interpolation is needed if the signals are non-periodic. From three different frequency bands of PSD features were extracted: very low frequency (VLF) band (0–0.04 Hz), low frequency (LF) band (0.04–0.15 Hz) related to the sympathetic activity, and high-frequency (HF) band (0.15–0.4 Hz) related to the parasympathetic activity of ANS [76]. Features in the non-linear domain were calculated with Poincare plots. The following 26 features were extracted (abbreviations in brackets):

- Time-domain features: Mean RR interval length (meanRR), maximum RR interval length (maxRR), minimum RR interval length (minRR), range of RR interval length (rangeRR), standard deviation of RR interval lengths (SDNN), mean of 5-min standard deviation of RR intervals (SDANNIndex), maximum heart rate (maxHR), minimum heart rate (minHR), average heart rate (meanHR), standard deviation of heart rate (SDHR), square root of the mean squared difference of successive RR intervals (RMSSD), number of interval differences of successive RR intervals greater than 50 ms (NN50), percentage of successive/adjacent RR intervals differing by 50 ms (pNN50);
- Frequency-domain features: Very low frequency power (VLF), low frequency power (LFpower), high frequency power (HFpower), total power (Totalpower), percentage value of very low frequency power (pVLF), percentage value of low frequency power (pLF), percentage value of high frequency power (pHF), normalized low frequency power (LFnorm), normalized high frequency power (HFnorm), ratio of low and high frequency (LFHF_ratio);
- Non-linear domain features: Standard deviation of instantaneous (short term) beat-to-beat R-R interval variability (SD1), standard deviation (SD) of the long term R-R interval variability (SD2), ratio of standard deviation 1 and standard deviation 2;

After extraction, features were labeled for supervised machine learning (see Figure 4). In total, data from 27 out of 30 subjects were available for feature extraction from the wristband. To provide comparability, the missing three subjects were not considered in the case of the ECG. Overall, the number of “non-drowsy” and “drowsy” instances for the wristband are 14,627 and 3987 and for ECG 24149 and 5845. It becomes clear that ECG contains more instances, which is due to a more accurate, higher-resolution measurement compared to the wristband.

### 4.4. Classification of Driver Drowsiness

**Figure 5:**
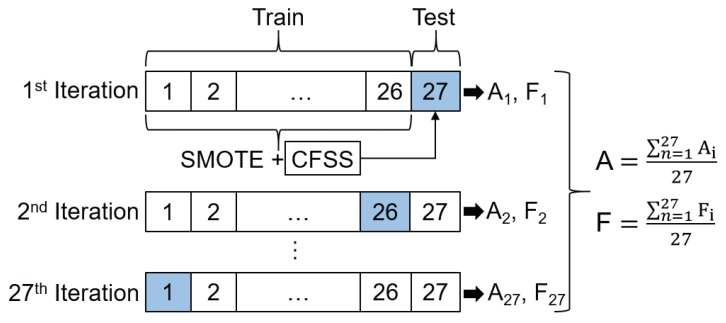
Graphic representation of Leave-One-Subject-Out Cross-Validation (LOSOCV) for 27 subjects: Oversampling of minority class in training set in each iteration with Synthetic Minority Oversampling Technique (SMOTE); Selection of best feature subset with Correlation-based Feature Subset Selection (CFSS) in training set and adjusting of features in testing set; calculation of average accuracy (A) and F-measure (F) (separately for both classes) across all iterations.

The ability to generalize for new users is a crucial issue for the establishment of systems for driver activity recognition. Thereby, the problem of inter-driver variance has to be taken into account, because physiological signals within persons, in our case, drivers of an automated vehicle, can differ to a great extent [85]. We apply a User-Independent Test (UIT) to deal with this issue. In the UIT, a Leave-One-Subject-Out Cross-Validation (LOSOCV) is performed. The data set for each subject is treated as testing data once. Since, in the present case, data from 27 participants were collected, in each LOSOCV-iteration, 26 participants are used for training and the remaining 27th for testing. The prediction results are then averaged over all subjects (see Figure 5). In comparison to the UIT, a User-Dependent Test (UDT) will be performed additionally in the form of 10-fold stratified Cross-Validation (CV) to obtain the overall classification accuracy and to decrease the effect of inter-driver variance. Stratified cross-validation was utilized because each fold reflects the class distribution in the original data set. In terms of our classification problem and the present class imbalance, this ensures the same proportion of drowsy and non-drowsy samples in each cross-validation run. Moreover, it reduces both bias and variance compared to regular k-fold cross-validation, where the data set is only randomly divided into k folds [86].

#### 4.4.1. Class Balancing and Feature Selection

To counteract class imbalance, the number of instances in the two classes has been adjusted using the “Synthetic Minority Oversampling Technique (SMOTE)” [87]. During LOSOCV and 10-fold cross-validation, SMOTE was applied to the training set in each iteration before the classifier was trained (see Figure 5).

To reduce the feature space, feature selection on each training set was performed in each iteration step after oversampling. For this purpose, Correlation-based Feature Subset Selection (CFSS) was applied. CFSS searches for a subset of features that is highly correlated to the output class, yet its features unrelated to each other. Thereby, feature-to-feature correlation is reduced, and feature-to-class correlation increased. The criterion is defined using the Pearson correlation coefficient. CFSS is not a search method; rather, it proposes a metric to evaluate a certain feature subset. Best-first was used as search algorithm. The predictive ability of each feature is used to evaluate the worth of a subset, in consideration of the redundancy between all features. [88]. Depending on the features in the obtained subset, in each iteration, the corresponding features were adjusted in the corresponding test data set (see Figure 5).

#### 4.4.2. Performance Measures

Concerning performance measures, accuracy might be as one of the traditional measures suitable but is not when dealing with unbalanced data because its focus is more on the majority classes than on the minority ones [89]. Thus, F-measure will be used additionally. For both UDT and UIT, accuracy and F-measure were calculated and averaged across all iterations (see Figure 5). With regard to the presented binary classification problem, it is, of course, important to correctly detect when the driver is in a drowsy state. From the customer’s point of view, however, it is also crucial to correctly detect when the driver is in a non-drowsy state, so as not to irritate with unnecessary drowsiness warnings. Concerning a standard confusion matrix with the values for “True Positive (TP)”, “True Negative (TN)”, “False Positive (FP)”, and “False Negative (FN)”, the formula for F-measure (see Equation (1)) does not take the True Negative (TN) values in account.

(1) $$F\text{-}measure=\frac{2TP}{2TP+FP+FN}$$

In the presented case, the correctly classified instances of the negative class, representing “drowsy”, would not be considered. For this reason, at each cross-validation iteration, the value for F-measure is calculated per class. For the “non-drowsy”, i.e., positive class, this is further referenced as F1 and for the “drowsy”, i.e., negative class as F2. An average value is then presented for both F1 and F2 across all subjects. Therefore, F2 is the crucial measure for the detection of drowsiness, since its value represents how many “drowsy” instances were correctly classified as “drowsy”.

## 5. Results

The machine learning procedure, including oversampling and feature selection, was performed with the Weka machine learning library implemented in Java [90].

### 5.1. Selected Features

The feature selection procedure using CFSS was performed on the training data. In the next step, the features in the testing data were adjusted accordingly. This was done before the actual training and testing the machine learning model. Table 5 presents all selected feature subsets in CFSS for UDT and UIT for both devices. The total number of available subsets equals the number of iterations in each cross-validation: 10 for UDT (10-fold stratified CV) and 27 for UIT (LOSOCV with 27 subjects).

When looking closer at the selected feature subsets for the UDT, it can be seen that they consist only of time-domain features. However, the subsets of both devices do not contain any identical features. In the case of the wristband, a total of five different features are selected. Each subset includes meanRR and meanHR. In the case of ECG, maxRR and minRR appear in eight subsets. Furthermore, maxHR and minHR were selected. The low number of subsets can be related to the fact that the data of all subjects were randomized and evenly divided into ten folds in a stratified way to counter inter-driver variance between the subjects.

In contrast, when looking at the selected feature subsets for UIT and comparing them with those for the UDT, 11 different feature subsets were selected with data from the wristband, which are almost four times as many as for UDT. In addition to time-domain features, in CFSS also features from the frequency- and non-linear domain were selected in the feature subsets. It becomes clear that the inter-driver variance has an influence on the choice of features if only a single person is removed from the data set. This can also be recognized for ECG where twice as many feature subsets exist but, as for the UDT, containing again only features from the time-domain and therein min/max values of the RR and HR signals.

In general, for both the UDT and UIT, the majority of selected feature subsets for wristband and ECG mainly consist of time-domain features. The importance of the features for the present case can be ranked in descending order as follows: time-domain, frequency-domain, non-linear domain.

### 5.2. Classification Results

Several classifiers of the Weka machine learning library were applied with the data collected during the simulator study. The models were not fine-tuned, but the default parameter values used, that were preset in the Weka machine learning library [90]. Models from different categories were compared with each other in terms of their performance in the presented classification problem. From the category of tree classifiers, Random Forest (RF) (100 trees), Random Tree (RT), and Decision Stump (DS) were chosen. In terms of rule-based classifiers, a Decision Table (DT) (search algorithm: Best first, evaluation measure: root-mean-square error (RMSE)) was applied. The K-Nearest Neighbor (KNN) algorithm (no distance weighting; number of neighbors: 1, search algorithm: brute-force, distance function: Euclidean) from the group of lazy learners and from the group of Bayesian classifiers a Bayesian Network (BN) (estimator: simple estimator, search algorithm: K2), and Naive Bayes (NB) classifier were tested. A Support Vector Machine (SVM) classifier (kernel: polynomial, C: 1) represented a function-based-classifier. A Multilayer Perceptron (MLP) (batch size: 100, hidden layers: (number of features + number of classes)/2, learning rate: 0.3, momentum: 0.2) from the group of neural networks was applied. For each of the models presented, the exact same procedure for testing was carried out, i.e., a 10-fold stratified CV in the UDT and a LOSOCV in the UIT, as described above.

Table 6 shows the results of UDT and UIT, for both devices and all models tested, with the respective values for accuracy and F-measure. Focusing on the accuracy of the UDT, it can be seen that ECG data produced better results on all tested classifiers except NB. These differences are, in some cases, more pronounced as in BN, SVM, DS, and DT, but for KNN, RF, and RT, the difference is only a few percentage points. For the wristband, the highest accuracy of 92.13% was achieved with KNN, 91.58% with RF, and 90.02% with RT. For ECG, the classifiers RF and RT performed best with an accuracy of 97.37%, as well as BN with 96.85% and DT with 91.18%. In terms of F-measure, it is noticeable that the values for F1 and F2 diverge more in the wristband. Looking at RF and RT, the values for F1 are 0.94 and 0.93, and the values for F2 are 0.82 and 0.79. For ECG, these are 0.98 and 0.94 each. In general and concerning F1, the values of the wristband are slightly lower but comparable to ECG. Except for DS and MLP, which has higher values for F2 than for ECG, the differences between the F1 and F2 are larger. redFor ECG, the values for F1 and F2 are very high and at a similar level for BN, KNN, RF, RT, and DT. This speaks for a low number of false positives and false negatives and for an equally satisfying classification in both classes. The assessment of drowsy instances as really drowsy has shown to be more difficult for the models when working with data from the wristband what can be referred to lower values for F2 and a higher number of false negatives, compared to ECG.

With regard to UIT and except SVM (65.64% accuracy), overall lower classification accuracies were achieved when compared to UDT. In addition to NB (66.74%), SVM (65.64%), and MLP (25.84%) yielded better results with data from the wristband. All other models achieved higher results with HRV from ECG data. However, the differences between the two devices are not as high as in the UDT case. DS achieved the highest accuracy with 73.39% and was the only model >70%. Regarding ECG data, the classifiers NB (41.84%), SVM (40.01%), and MLP (25.84%) did not reach the threshold of 50%. In terms of accuracy, DS scored the best with ECG at 78.94%. Focusing on the values of F1 and F2, it is noticeable that in F1 for both devices, the values are lower compared to the UDT. In the UIT, the maximum F1 score for both the wristband and ECG was achieved with DS (0.82). In contrast, values for F2 are significantly lower. DS achieved a maximum of 0.65 with data from the wristband and DT with ECG data 0.37. In general, the number of false negatives in the “drowsy” class is way higher than the number of false positives in the “non-drowsy” class, which resulted in the low values for F2. To get a better impression of the classification results, Table 7 shows exemplary UIT classification results for four individual subjects (3, 4, 21, and 27) and five selected classifiers (BN, KNN, RF, RT, and DT) for wristband and ECG. The values for F2 show again that the classification of “drowsy” instances is more challenging than the “non-drowsy” ones. Generally speaking, it also becomes clear that the performance depends very much on the individual subject, which reflects the strong influence of the inter-driver variance. Depending on the model, but also on the type of data, strong fluctuations in performance exist already within this small extract from the data set.

### 5.3. Discussion and Limitations

Focusing on the results of the two devices in general, regardless of whether a UDT or UIT was performed, it is noticeable that with ECG data, slightly better results for the majority of the tested models were achieved. However, focusing on RQ2, it should be noted that with the data of the consumer device, albeit not for all models, the results are comparable and at a similar level as compared to the more intrusive medical-grade device in the in-vehicle setting. Thus, for the present application, no more complex ECG measurement would have to be applied. Instead, the much less intrusive sensor of the wrist-worn smart wearable would suffice. High accuracies (KNN: 92.13%, RF: 91.58%, RT: 90.02%) as well as values for F-measure, and especially with KNN (F1: 0.95, F2: 0.83), were achieved in the UDT with data from the wristband. Concerning RQ1, this speaks for the use of physiological data, i.e., HRV, from a wristband as single data source for driver drowsiness detection with user-dependent models. Results from UDT are significantly higher than in the UIT (RQ3), which may reflect the more considerable influence of inter-driver variance that could have been better accounted for a more extensive data set. The classification of the “drowsy” class turned out to be particularly critical at UIT, which can be recognized by the low values of F2 for both devices. The maximum achieved score for F2 was 0.65 with wristband data and 0.37 with ECG data. This indicates the challenges that still need to be addressed before a robust commercial warning system can be developed. In comparison, UIT and UDT achieved high F1 values. From the customer’s point of view, this is of crucial importance, as the driver does not want to be irritated by false drowsiness warnings. Moreover, data from more realistic environments has to be collected [91]. Vibrations in a real car that could influence the data recording were absent in the simulator. To be able to counteract the class imbalance, cost-sensitive classification should also be considered, in which the values of TP, FP, TN, and FN carry different weightings. Throughout the use, the system could transit from a user-independent model to a user-dependent one and adapt to the user. In terms of the ground truth, the question arises, whether it is sufficient to use drowsiness ratings that apply to 5-min intervals, or ratings at much shorter intervals are needed. During the transition to the adjacent 5-min interval, possible changes in the course of drowsiness ratings may not be reflected by the physiological signal. The applied models were used “off-the-shelf”, and no hyper-parameters were tuned. The aim was to identify which standard machine learning models are suitable for the proposed classification problem. Fine-tuning of the most promising models could then increase performance further. Since the focus in this work was on a specific type of feature selection (CFSS) and class balancing, other methods should be considered and compared.

Concerning the previous studies on driver drowsiness detection using wrist-worn wearable devices, a one-to-one comparison with the proposed methodology is not possible because of the use of different data sets. The involvement of multiple sensors makes the systems more intrusive [47] and complex [70], whereas the proposed model only uses a wristband. In other studies, new wrist-worn wearables for recording physiological data were developed [68,69]. In contrast to the device used in this work, these devices are not yet ready for the market, which makes a future and large-scale use in the vehicle difficult. The use of motion sensors in the wearables to evaluate the steering behavior will be more difficult in the future [66,67], since the degree of automation will steadily increase. By using physiological data from wearables, as in the previous case as the sole data source that is continuously measured, this problem no longer exists. Except for one study [47], all others used SVM for classification. The majority achieved very high accuracies of around 98%. In the present case, lower accuracies were achieved with SVM, especially with the UDT, where the wristband data only achieved an accuracy of 30%, which can be due to several reasons, such as a different database, labels or feature extraction methodology. With our proposed approach with a KNN classifier, around 92% could be achieved by using only physiological data of the wristband. The proposed methodology was implemented to show and discuss the feasibility of using solely physiological data from a wrist-worn wearable device for driver drowsiness detection. From the results, it can be seen that drowsiness can be effectively detected. The gained knowledge can serve as a starting point for further research in this area.

## 6. Conclusions

To explore the applicability of wrist-worn wearable devices for driver drowsiness detection, heart rate data from a wristband, as well as an ECG device during a simulator study with level-2 automated driving, were collected. The drowsiness level of the driver was determined based on a combination of video ratings and detected micro-sleep events. HRV features were extracted using a sliding-window approach and labeled with the corresponding drowsiness level. The performance of several machine learning classifiers was compared. Results depict that in the UDT, better results were achieved because of the reduced influence of inter-driver variance. With a value of 92.13%, KNN had the highest accuracy with data from the wristband. With ECG data, 97.37% was reached with RT and RF. In the UIT, especially, the drowsy instances, were more challenging to classify. The wristband achieved 73.39% accuracy with a DS. The maximum for ECG was 78.94%. In general, for both UDT and UIT, the obtained results with data from wristband and ECG are comparable. The results of our proposed methodology show that driver drowsiness detection using exclusively physiological data (HRV) from a standalone wrist-worn wearable device in combination with a machine learning model is feasible. Moreover, instead of producing a new device, the models were developed for a device available on the market that qualifies the shown method for potentially any consumer device, indicating that binary classification of drowsiness is feasible with today’s technology on the consumer electronics market. Since metrics such as gyroscope or accelerometer will be more challenging to apply for driver drowsiness detection in future automated driving, thereby, our presented method would draw more attention.

The acquired knowledge can be useful for further studies in the area of driver drowsiness detection, and the advancement of vehicle-to-driver interaction concepts, furthermore, for safety-critical tasks in the area of automated driving.

### Future Work

In future work, existing models improved. For feature extraction, other methods and in the present case, different increments and window sizes on the sliding window approach will be examined, and the feature space enlarged. For feature selection and class balancing, other methods than the presented ones will be considered. Since a combination of video ratings and micro-sleep events served as ground truth, in the future, the usage of driver self-ratings is planned for a comparison of classification accuracies. For an increasing generalization ability, the data set has to be enriched, and above all, with drowsy samples. Data from more users and realistic study environments will be collected. Furthermore, a prototype for real-time detection will be developed.

## Figures and Tables

**Table 1:** Scale for video ratings with indicators per level [13].

Drowsiness Level	Indicators
**1** not drowsy	appearance of alertness present; normal facial tone; normal fast eye blinks; short ordinary glances; occasional body movements/gestures
**2** slightly drowsy	still sufficiently alert; less sharp/alert looks; longer glances; slower eye blinks; first mannerisms as: rubbing face/eyes, scratching, facial contortions, moving restlessly in the seat
**3** moderately drowsy	mannerisms; slower eye lid closures; decreasing facial tone; glassy eyes; staring at fixed position
**4** drowsy	eyelid closures (1–2 s); eyes rolling sideways; rarer blinks; no proper focused eyes; decreased facial tone; lack of apparent activity; large isolated or punctuating movements;
**5** very drowsy	eyelid closures (2–3 s); eyes rolling upward/sideways; no proper focused eyes; decreased facial tone; lack of apparent activity; large isolated or punctuating movements;
**6** extremely drowsy	eyelid closures (4 s or more); falling asleep; longer periods of lack of activity; movements when transition in and out of dozing;

**Table 2:** Categorization and allocation of the 201 detected micro-sleep events to the corresponding level of drowsiness based on the eyelid closure time.

Drowsiness Level	Eyelid Closure Time	Micro-Sleep Events
4: drowsy	1 ≤ seconds < 2	89
5: very drowsy	2 ≤ seconds < 4	69
6: extremely drowsy	seconds ≥ 4	43

**Table 3:** Adjustment of observer ratings after integration of detected micro-sleep events with number of occurrences for each case; n.a.: no observer rating available.

Observer Rating	Adjusted Rating	Number of Occurrences
1	4	1
2	4	3
2	5	4
2	6	1
3	4	2
3	5	5
4	5	3
5	6	1
6	4	1
n.a.	6	2

**Table 4:** Distribution of number of video ratings in absolute numbers across grouped drowsiness levels before and after integration of micro-sleep events.

Class	Video Ratings	Video Ratings + Micro-Sleep Events
non-drowsy (level 1–3)	212	196
drowsy (level 4–6)	32	50

**Table 5:** Selected Feature Subsets in CFSS for User-Dependent Test (UDT) (10 iterations in 10-fold Cross Validation (CV)) and User-Independent Test (UIT) (27 iterations in LOSOCV) with number of occurrences for wristband and ECG.

UDT	
Wristband	8× meanRR, meanHR 1× meanRR, meanHR, RMSSD 1× meanRR, meanHR, RMSSD, NN50
ECG	3× maxRR, minRR, maxHR, minHR 2× maxHR, minHR 5× maxRR, minRR
**UIT**	
Wristband	5× meanRR, meanHR, SD1 1× meanRR, maxRR, SD1, LFpower 1× meanRR, maxRR, meanHR 5× meanRR, meanHRV, RMSSD 9× meanRR, meanHR 1× meanRR, meanHR, HFpower 1× meanRR, meanHR, pNN50, SD1 1× meanRR, meanHR, RMSSD, LFpower 1× meanRR, meanHR, SD2 1× meanRR, meanHR, Totalpower 1× meanRR, minHR, meanHR, RMSSD, NN50
ECG	8× maxRR, minRR, maxHR, minHR 5× maxRR, minRR, rangeRR, maxHR, minHR 3× maxRR, maxHR 1× maxHR, minHR 6× minRR, minHR 4× maxRR, minRR

**Table 6:** Classification results with performance measures for UDT/UIT, Wristband/ECG and for all tested classifiers. The abbreviations stand for accuracy (A), F-measure for “non-drowsy” class (F1) and F-measure for “drowsy” class (F2). The best performing models are printed in boldface.

**UDT**	**Wristband**			**ECG**		
**Model**	**A**	**F1**	**F2**	**A**	**F1**	**F2**
BN	79.59	0.86	0.62	**96.85**	**0.98**	**0.92**
NB	68.39	0.79	0.32	53.88	0.66	0.26
SVM	29.27	0.72	0.32	54.39	0.66	0.28
KNN	**92.13**	**0.95**	**0.83**	97.34	0.98	0.94
RF	**91.58**	**0.94**	**0.82**	**97.37**	**0.98**	**0.94**
RT	**90.02**	**0.93**	**0.79**	**97.37**	**0.98**	**0.94**
DS	77.60	0.86	0.36	81.36	0.89	0.21
DT	80.61	0.87	0.65	**97.18**	**0.98**	**0.93**
MLP	61.94	0.69	0.46	64.43	0.71	0.30
**UIT**		**Wristband**			**ECG**	
**Model**	**A**	**F1**	**F2**	**A**	**F1**	**F2**
BN	57.77	0.69	0.19	70.86	0.77	0.10
NB	66.74	0.80	0.57	41.84	0.63	0.20
SVM	65.64	0.79	0.26	40.01	0.61	0.28
KNN	55.44	0.71	0.12	65.71	0.75	0.14
RF	62.36	0.74	0.20	70.64	0.79	0.13
RT	63.16	0.72	0.19	68.88	0.76	0.21
DS	**73.39**	**0.82**	**0.65**	**78.94**	**0.83**	**0.17**
DT	64.28	0.73	0.15	**76.14**	**0.73**	**0.37**
MLP	43.48	0.57	0.42	25.84	0.49	0.22

**Table 7:** Exemplary classification results of UIT for selected classifiers and subjects with performance measures for both wristband and ECG.

Participant		3			4			21			27		
Model	Device	A	F1	F2	A	F1	F2	A	F1	F2	A	F1	F2
BN	Wristband	59.90	0.75	0.00	20.45	0.32	0.04	52.30	0.65	0.27	18.02	0.17	0.20
	ECG	51.73	0.59	0.41	36.57	0.54	0.00	77.06	0.87	0.00	85.49	0.92	0.51
KNN	Wristband	58.17	0.73	0.05	22.30	0.37	0.00	62.20	0.77	0.00	42.55	0.60	0.01
	ECG	63.41	0.68	0.58	42.93	0.59	0.07	74.56	0.85	0.04	78.65	0.86	0.26
RF	Wristband	57.37	0.73	0.02	53.16	0.14	0.68	63.65	0.78	0.00	47.43	0.64	0.04
	ECG	66.62	0.77	0.36	42.29	0.54	0.23	72.40	0.84	0.02	83.49	0.91	0.00
RT	Wristband	56.74	0.72	0.02	40.90	0.52	0.24	63.29	0.76	0.00	47.06	0.63	0.04
	ECG	67.27	0.78	0.36	42.29	0.54	0.23	74.81	0.86	0.02	87.49	0.93	0.00
DT	Wristband	49.60	0.66	0.00	34.20	0.04	0.50	58.09	0.74	0.00	33.29	0.43	0.19
	ECG	38.25	0.00	0.55	60.57	0.40	0.71	87.49	0.93	0.00	87.48	0.93	0.00
